# Promoting Long-Term Survival of Insulin-Producing Cell Grafts That Differentiate from Adipose Tissue-Derived Stem Cells to Cure Type 1 Diabetes

**DOI:** 10.1371/journal.pone.0029706

**Published:** 2011-12-28

**Authors:** Shuzi Zhang, Hehua Dai, Ni Wan, Yolonda Moore, Zhenhua Dai

**Affiliations:** Department of Microbiology and Immunology, Center for Biomedical Research, University of Texas Health Science Center, Tyler, Texas, United States of America; La Jolla Institute for Allergy and Immunology, United States of America

## Abstract

**Background:**

Insulin-producing cell clusters (IPCCs) have recently been generated in vitro from adipose tissue-derived stem cells (ASCs) to circumvent islet shortage. However, it is unknown how long they can survive upon transplantation, whether they are eventually rejected by recipients, and how their long-term survival can be induced to permanently cure type 1 diabetes. IPCC graft survival is critical for their clinical application and this issue must be systematically addressed prior to their in-depth clinical trials.

**Methodology/Principal Findings:**

Here we found that IPCC grafts that differentiated from murine ASCs in vitro, unlike their freshly isolated islet counterparts, did not survive long-term in syngeneic mice, suggesting that ASC-derived IPCCs have intrinsic survival disadvantage over freshly isolated islets. Indeed, β cells retrieved from IPCC syngrafts underwent faster apoptosis than their islet counterparts. However, blocking both Fas and TNF receptor death pathways inhibited their apoptosis and restored their long-term survival in syngeneic recipients. Furthermore, blocking CD40-CD154 costimulation and Fas/TNF signaling induced long-term IPCC allograft survival in overwhelming majority of recipients. Importantly, Fas-deficient IPCC allografts exhibited certain immune privilege and enjoyed long-term survival in diabetic NOD mice in the presence of CD28/CD40 joint blockade while their islet counterparts failed to do so.

**Conclusions/Significance:**

Long-term survival of ASC-derived IPCC syngeneic grafts requires blocking Fas and TNF death pathways, whereas blocking both death pathways and CD28/CD40 costimulation is needed for long-term IPCC allograft survival in diabetic NOD mice. Our studies have important clinical implications for treating type 1 diabetes via ASC-derived IPCC transplantation.

## Introduction

Pancreatic islet transplantation holds much promise for the cure of type 1 diabetes as transplantation of cadaveric islets is already conducted in the clinic to treat patients with type 1 diabetes. However, the scarcity of human donor islets remains a major obstacle to widespread islet transplantation. It is therefore compelling to search for alternative sources of islets. Embryonic stem cells originally have been exploited as a source for β cells due to their tremendous differentiation potential. Previous studies have shown that insulin-secreting cells are indeed generated from embryonic stem cells [1], [2], [3], [4], [5], [6], [7]. However, their application in translational medicine could be limited because of ethical and legal concerns. Therefore, adult mesenchymal stem cells have recently been studied to generate β cells. Previous studies have shown that insulin-producing cells can be generated from bone marrow cells [8], hepatic [9] and pancreatic stem cells [10]. Nevertheless, the limited sources and invasive procedures have hampered their progress.

Adipose tissue has recently gained much attention as a prime source of mesenchymal stem cells that can differentiate into the cells of mesodermal origin, including insulin-producing cell clusters (IPCCs) [11]. The simple surgical procedure, easy accessibility, uncomplicated isolation and tissue abundance make adipose tissue a most attractive source of mesenchymal stem cells for researchers [11], [12], [13]. Indeed, recent studies have shown that IPCCs can differentiate from both human and murine adipose tissue-derived stem cells (ASCs) [14], [15], [16], [17]. Moreover, transplantation of ASCs over-expressing Pdx1 gene [18], [19] or IPCCs generated from ASCs in vitro [16], [17] restored normoglycemia in chemical-induced diabetic mice, although it was not known how long IPCC graft survival could last, suggesting that ASCs or ASC-derived IPCCs may be potentially utilized to treat human type 1 diabetes. However, it remains unknown how long IPCC grafts survive upon transplantation and how their long-term survival can be induced in diabetic recipients.

In this study, we generated IPCCs that differentiated from murine ASCs in vitro and investigated their survival after transplantation in diabetic mice. The aim of this study is to induce long-term IPCC graft survival in a preclinical animal model, because their long-term survival is critical for the cure of type 1 diabetes. Previous studies have focused on other important issues at the early stage of IPCC studies, including the generation of IPCCs in vitro, their temporary functions in vivo, and their immunogenicity. The novelty of this study lies in the first induction of long-term IPCC graft survival in diabetic mice, including autoimmune-prone NOD mice, by blocking both IPCC cell death and T cell costimulation.

## Results

### In vitro differentiation of ASCs into IPCCs

**Figure 1:**
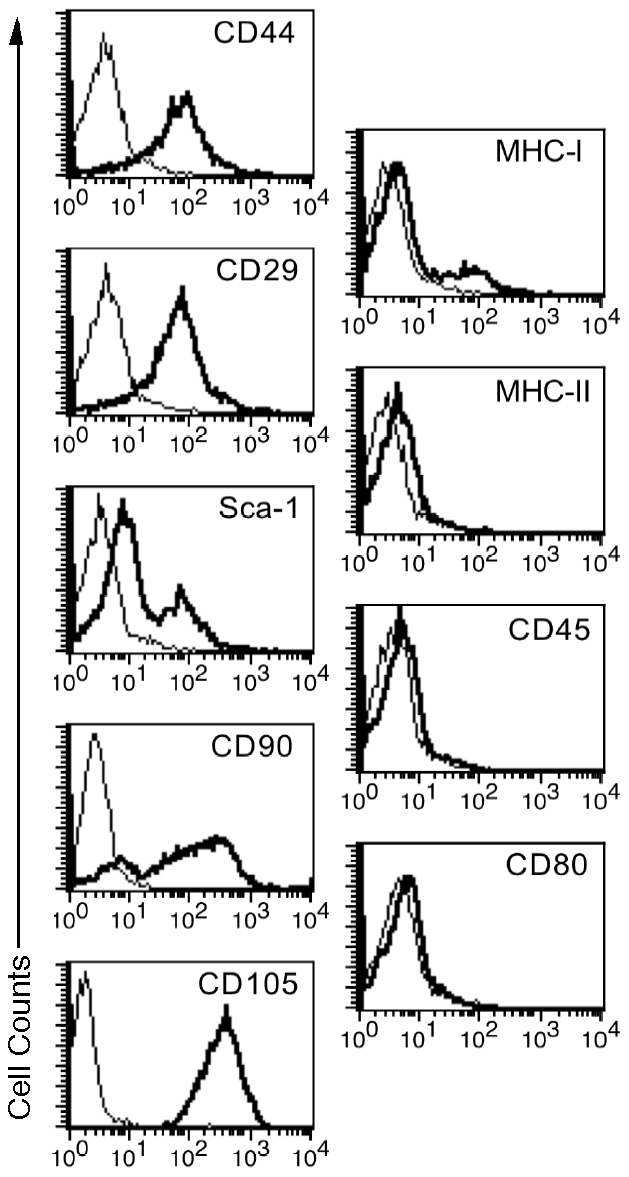
In vitro characterization of ASCs for their markers. Expanded ASC clone at passage 4 was assessed by flow cytometric analysis. Expanded ASCs highly expressed CD29, CD44, CD90, CD105 and Sca-1 while their MHC class-I expression was low. They did not express MHC class-II, CD45 and CD80 at all. One representative of three separate experiments is shown.

**Figure 2:**
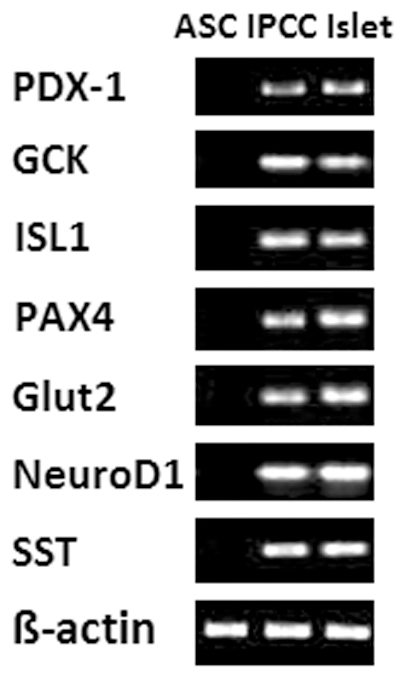
Gene expression of pancreatic endocrine and development markers by ASC-derived IPCCs. Expression of genes by day-10 IPCCs that are related to pancreatic development, hormones and glucose sensing was analyzed by RT-PCR. One representative of three independent experiments is shown.

**Figure 3:**
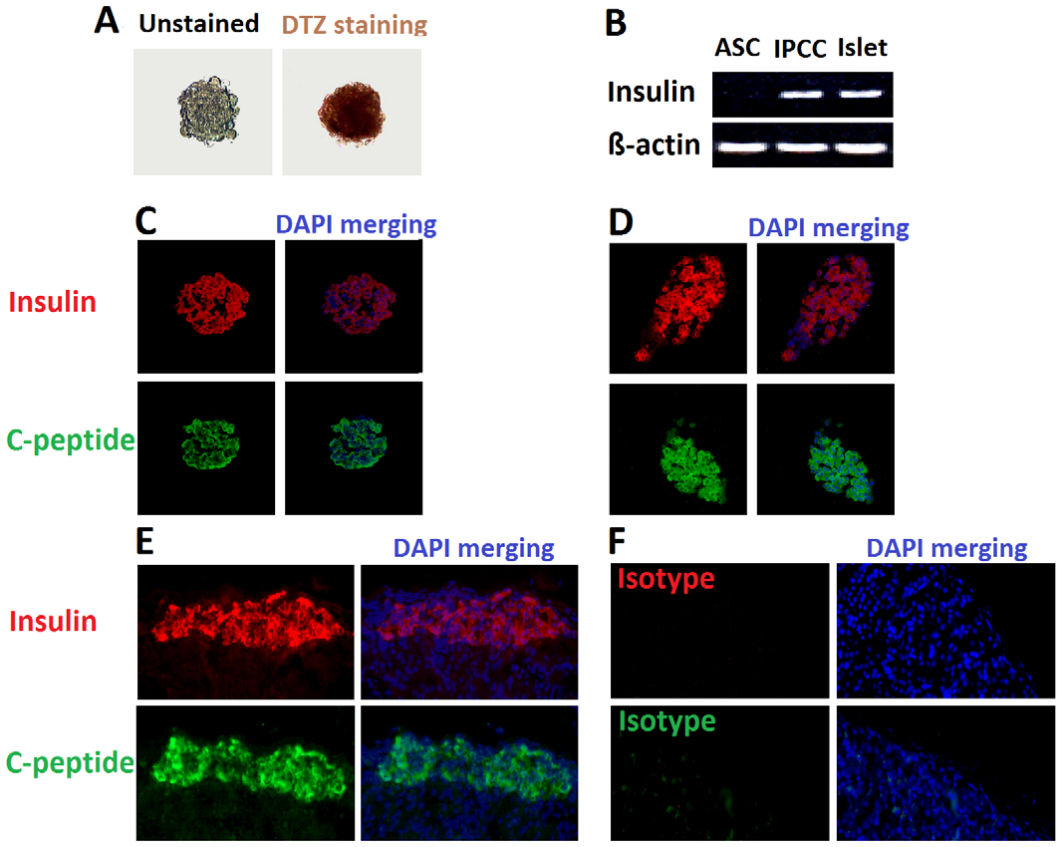
DTZ staining, gene expression and immunofluorescence analysis of IPCCs. (**A**): Day-10 IPCCs were stained with dithizone (DTZ), a agent that selectively stains pancreatic β cells because of their high zinc content. (**B**): Insulin and C-peptide gene expression by RT-PCR. (**C**): Day-10 IPCCs were stained for insulin and C-peptide without transplantation. (**D**) Fresh islets were stained for insulin and C-peptide without transplantation. (**E**): IPCC grafts under the kidney capsule were stained with insulin and C-peptide five days after their transplantation. (**F**): Isotype Ab staining for IPCC grafts. Cell nuclei were stained with DAPI. One representative of three separate experiments is shown.

ASCs were isolated from the fat pads of C57BL/6 mice as described previously [16], [20]. Although newly isolated ASCs had a heterogeneous phenotype in the initial culture, a single fibroblastoid cell population was expanded following the subsequent cultures. The homogeneity of ASCs at passages 4–6 was confirmed by FACS analysis showing that they highly expressed CD29, CD44, CD90, CD105 and Sca-1 surface markers [16], as shown in Figure 1. They also expressed low MHC-class-I while lacking MHC-class-II, CD45 and CD80. Day-10 IPCCs highly expressed many genes related to pancreatic endocrine development and glucose sensing (Figure 2). The IPCCs were stained positively for DTZ, a zinc-chelating agent that specifically stains pancreatic β cells (Figure 3A). RT-PCR also showed that IPCCs and islets express insulin mRNA while undifferentiated ASCs do not (Figure 3B). Immunofluorescence staining demonstrated that both IPCCs (Figure 3C) and freshly isolated islets (Figure 3D) abundantly express insulin and C-peptide before transplantation. Moreover, IPCC grafts also highly expressed insulin and C-peptide (Figure 3E) compared to isotype control (Figure 3F) five days after they were transplanted under the kidney capsule of recipient mice. These findings confirmed that IPCCs express both insulin and C-peptide at both gene and protein levels. Finally, IPCCs, just like their islet counterparts, released significant amount of insulin upon in vitro-stimulation with high glucose, as shown in the supporting information (Figure S1), suggesting that ASC-derived IPCCs are fully functional.

### Blocking Fas and TNF signaling promotes IPCC graft survival in syngeneic recipients

**Figure 4:**
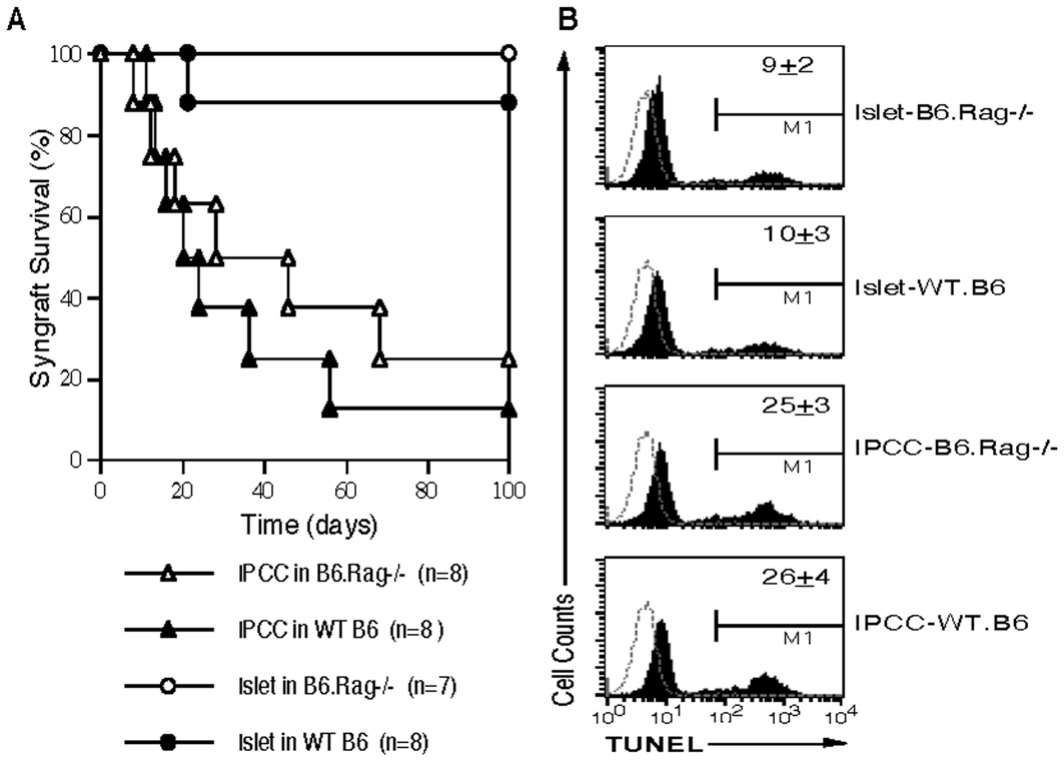
Impaired IPCC, but not islet, graft survival in syngeneic recipient mice. Islets freshly isolated from B6 donors or IPCCs that differentiated from B6-derived ASCs were transplanted under kidney capsule of either B6.Rag-/- or WT B6 recipient mice that were rendered diabetic by a single injection of streptozotocin before transplantation. Islet and IPCC syngraft rejection was observed (**A**). In separate groups, transplanted islets or IPCCs were retrieved and dissociated with trypsin-EDTA one week after transplantation. Cells were then stained with PE-conjugated PDX-1 and their apoptosis was measured by a TUNEL method (**B**). Histograms are gated on PDX-1-positive β cell population. One representative of three separate experiments is shown.

It remains unclear whether IPCC grafts, like freshly isolated islets, can survive forever in a syngeneic recipient. Either islets or ASC-derived IPCCs from B6 donors (H-2^b^) were transplanted to B6.Rag1-/- or WT B6 recipients. Surprisingly, we found that most IPCC syngrafts did not survive for long-term in both Rag1-/- and WT B6 recipients (Median survival time, MST = 37 and 22 days) while almost all of islet syngrafts survived for long-term in both recipients (Figure 4A), suggesting that ASC-derived IPCC grafts have a survival disadvantage over their freshly isolated islet counterparts, perhaps because of their passive cell death even in the absence of alloimmunity. We therefore retrieved IPCCs or islets and determined their cell apoptosis one week after their transplantation in syngeneic mice. As shown in Figure 4B, IPCC cells underwent much faster apoptosis than islet cells (percentage of TUNEL-positive cells: 25±3 or 26±4 vs. 9±2 or 10±3, both P<0.05).

**Figure 5:**
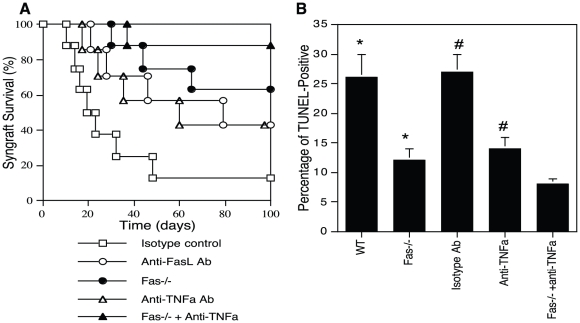
Blocking Fas and TNF receptor death pathways induces long-term IPCC graft survival in syngeneic WT mice. WT.B6-derived IPCCs were transplanted to WT B6 recipients that were treated with isotype Ab (Open square, n = 8), anti-FasL (Open circle, n = 7), or anti-TNFα Ab (Open triangle, n = 7). IPCCs derived from B6.Fas-/- ASCs also were transplanted to WT B6 recipients that were either untreated (Filled circle, n = 8) or treated with anti-TNFα Ab (Filled triangle, n = 8). IPCC syngraft rejection was then observed (**A**). IPCC graft cell apoptosis was determined by PDX-1 staining and a TUNEL method (**B**), as described in Figure 3. One representative of three separate experiments is shown.

We then investigated how to prevent the failure of IPCC grafts in syngeneic mice by transplanting IPCCs derived from either WT B6 or B6.Fas-/- donors to WT B6 recipients or treating recipients with anti-FasL or anti-TNFα blocking Ab. As shown in Figure 5A, most IPCC syngrafts (63%), which differentiated from Fas-/- ASCs, achieved long-term survival (MST>100 days) whereas 88% of IPCC syngrafts that differentiated from WT-derived ASCs failed to do so (MST = 22 days, Figure 4A). IPCCs that differentiated from Fas-/- ASCs were confirmed to be Fas-negative by FACS analysis (data not shown). Moreover, treatments with either anti-FasL or anti-TNFα blocking Ab in recipients that received Fas-replete IPCCs significantly prolonged IPCC syngraft survival compared with isotype Ab control (MST = 79 vs. 21 or 60 vs. 21 days, both P<0.05, Figure 5A). Importantly, blocking both Fas and TNF death pathways via anti-TNFα Ab treatment plus transplantation of Fas-deficient IPCCs induced long-term IPCC syngraft survival in 7 of 8 recipients. TUNEL analyses confirmed that either Fas deficiency or neutralizing TNFα significantly inhibited IPCC graft cell apoptosis in syngeneic recipients (Figure 5B). These data indicate that blocking Fas death signaling alone is capable of inducing long-term IPCC graft survival in majority of syngeneic recipients while blocking both Fas and TNF death pathways induces long-term IPCC graft survival in nearly all recipients.

### Blocking CD40-CD154 (CD40L) costimulation and death pathways induces long-term IPCC allograft survival

**Figure 6:**
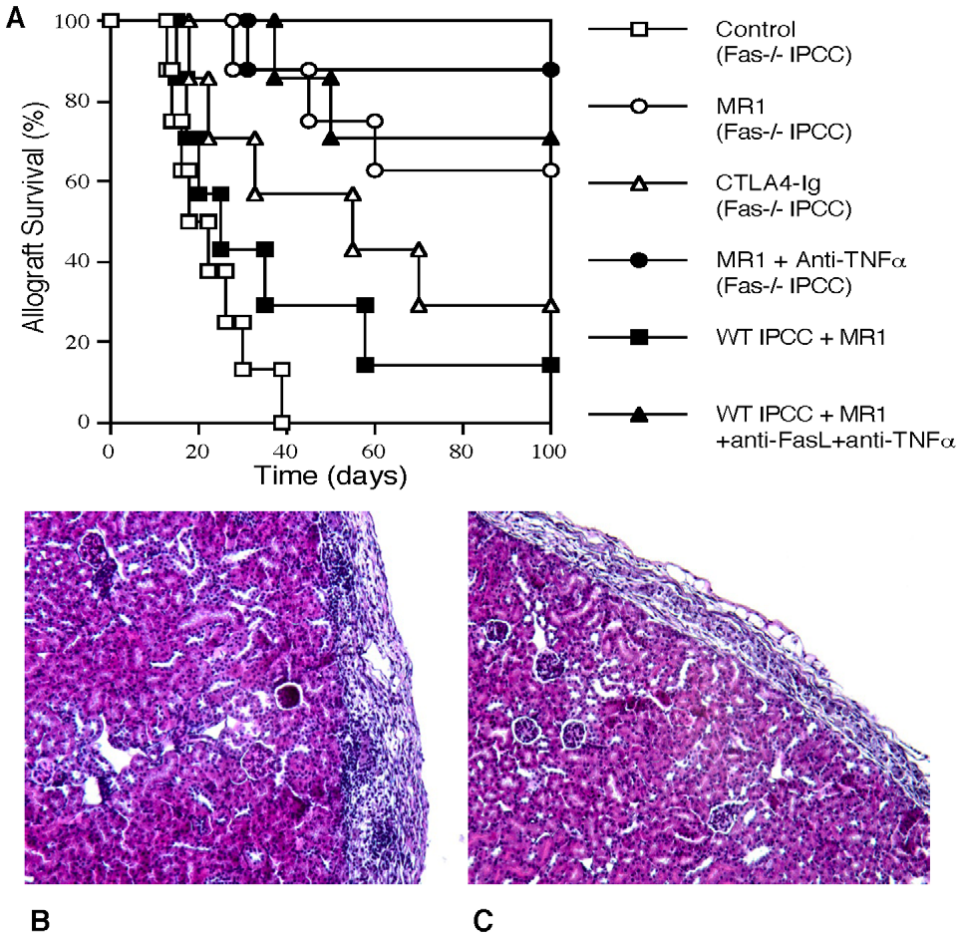
Blocking CD40-CD154 costimulation and Fas/TNF-receptor death pathways induces long-term IPCC allograft survival. IPCCs derived from B6.Fas-/- ASCs were transplanted to Balb/C recipients that were untreated (Open square, n = 8) or treated with MR1 (Open circle, n = 8), CTLA4-Ig (Open triangle, n = 7) or both MR1 and anti-TNFα Ab (Filled circle, n = 8). As a control, WT.B6-derived IPCCs also were transplanted to Balb/C recipients that were treated with MR1 alone (Filled square, n = 7) or MR1 + anti-FasL + anti- TNFα to block both CD154 costimulation and death pathways (Filled triangle, n = 7). IPCC allograft survival was observed (**A**). H&E staining of kidneys harboring Fas-/- IPCC allografts derived from an untreated control (**B**) or MR1+anti-TNF treated recipient (**C**) was performed two weeks after IPCC transplantation. (Magnification ×100).

Since IPCC recipients likely needs fat tissues from allogeneic donors as a source of ASCs, we investigated whether IPCC allografts would be quickly rejected. To minimize the passive cell death, IPCCs that differentiated from Fas-deficient ASCs isolated from B6.Fas-/- donors were transplanted to WT Balb/C recipients. Recipient mice were then treated with either MR1 (anti-CD154 Ab) or CTLA4-Ig to block CD40-CD154 or B7-CD28 costimulation. As shown in Figure 6A, all Fas-deficient IPCC allografts were rejected within 40 days (MST = 20 days), compared with 63% of Fas-deficient IPCC syngrafts that achieved long-term survival (Figure 5A), suggesting that IPCCs, like intact islets from donors, are immunogenic and subject to acute rejection. However, blocking CD40-CD154 costimulation induced long-term survival of Fas-deficient IPCC allografts in 63% of recipients (Figure 6A), indicating that CD40 blockade is sufficient to prevent IPCC allograft rejection mediated by alloimmune responses. Moreover, blocking CD28 also significantly prolonged IPCC allograft survival (MST = 55 vs. 20 days, P<0.05), albeit less effective than CD40 blockade (MST = 55 vs. >100 days, P<0.05). Blocking CD40 plus neutralizing TNFα further suppressed Fas-deficient IPCC allograft rejection with 88% of recipients achieving long-term survival. Finally, blocking CD40 and death pathways with triple Abs (MR1+ anti-FasL+ anti-TNFα) was confirmed to induce long-term Fas-replete IPCC allograft survival in most recipients (71%). As controls, isotype Abs did not alter IPCC allograft survival and Fas-replete IPCC allografts without any treatment were acutely rejected within 30 days (data not shown). The kidneys harboring IPCC grafts from some recipients with long-term graft survival were removed to confirm IPCC graft function in diabetic recipients. On the other hand, H&E staining demonstrated massive cell infiltration surrounding IPCC allografts in control group (Fig. 6B), but nearly no infiltration in MR1+anti-TNFα treated recipients (Fig. 6C) two weeks after IPCC transplantation. Taken together, these findings suggest that blocking CD40 and Fas/TNF-receptor death signaling is capable of inducing long-term IPCC allograft survival.

### Blocking CD40 or CD28 costimulatory pathway suppresses allograft-infiltrating T cell proliferation in vivo

**Figure 7:**
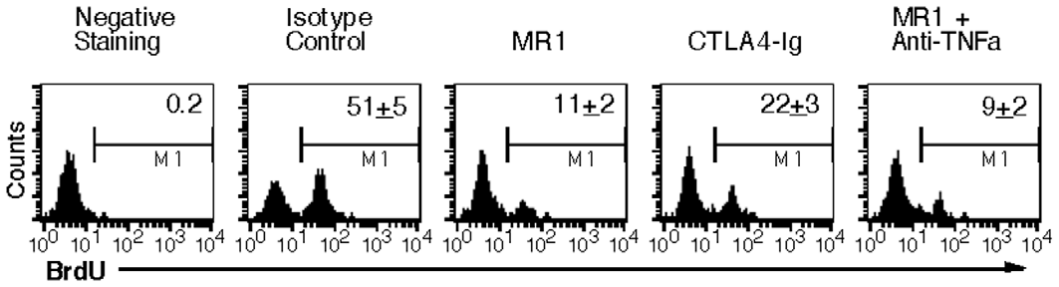
CD154 or CD28 costimulatory blockade inhibits the proliferation of T cells that infiltrate IPCC allografts. B6-derived IPCCs were transplanted to Balb/C recipients that were pulsed i.p. with 0.8 mg of BrdU six days after transplantation. 24 hours later, renal graft-infiltrating cells were isolated, stained with anti-CD3-PE and anti-BrdU-FITC Abs, and analyzed by FACS. Recipients were treated with MR1, CTLA4-Ig or anti-TNFα Ab. Histograms shown are gated on CD3+ cell population. One representative of three independent experiments is shown.

To further determine how the costimulatory blockade inhibits alloimmunity or IPCC allograft rejection, graft-infiltrating cells were isolated one week after IPCC transplantation and alloreactive T cell proliferation in vivo was measured by BrdU uptakes. As shown in Figure 7, treatment with either MR1 or CTLA4-Ig significantly reduced the percentage of BrdU-positive T cells (BrdU+: 11±2 vs. 51±5% or 22±3 vs. 51±5%, both P<0.05) although MR1 was more effective in the suppression of graft-infiltrating T cell proliferation than CTLA4-Ig (BrdU+: 11±2 vs. 22±3, P<0.05). Neutralizing TNFα plus MR1 did not further decrease the percentage of BrdU+ T cells compared with MR1 alone. Our data suggest that CD40 or CD28 costimulatory blockade suppresses IPCC allograft rejection by inhibition of alloreactive T cell proliferation.

### Blocking both CD40-CD154 and B7-CD28 costimulatory pathways is required for long-term IPCC allograft survival in diabetic NOD mice

**Figure 8:**
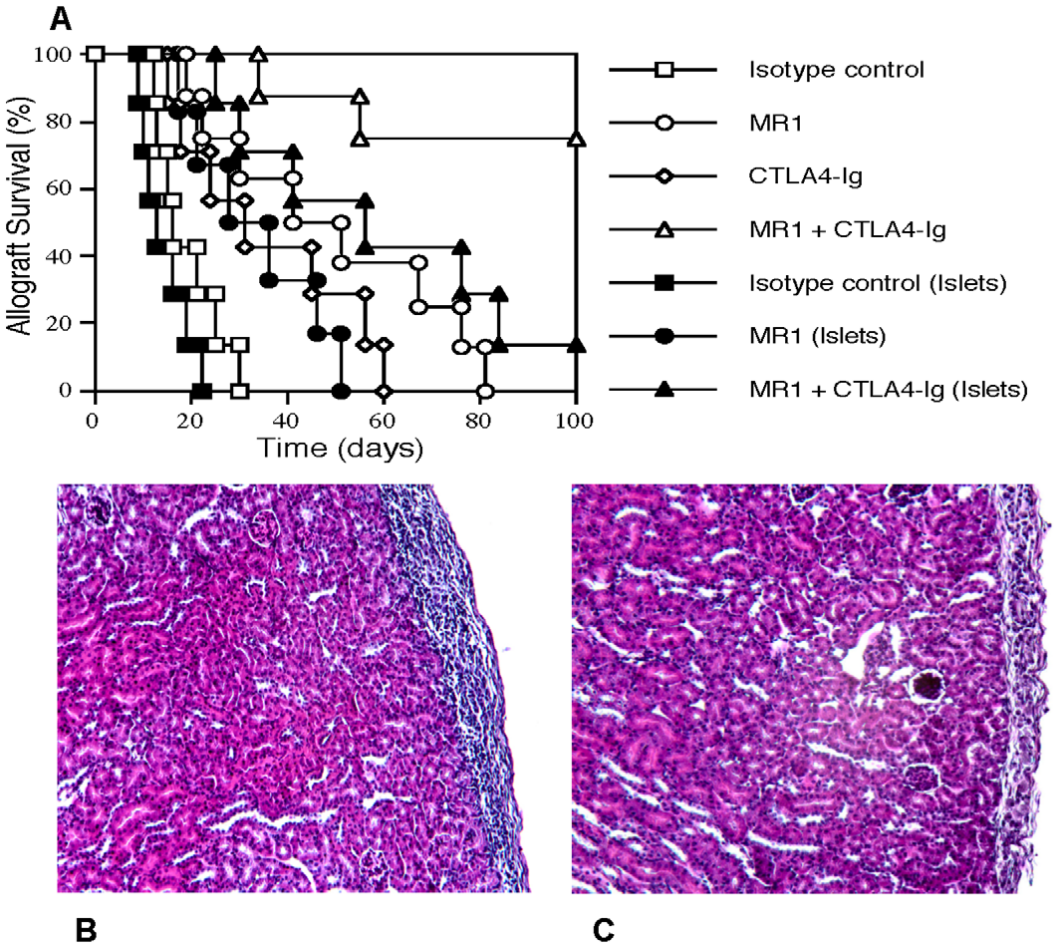
CD154/CD28 blockade induces long-term IPCC, but not freshly isolated islet, allograft survival in diabetic NOD mice. IPCCs that differentiated from Fas-deficient ASCs derived from MRL.MpJ.Fas-/- (H-2^k^) mice were transplanted to diabetic NOD mice that were treated with isotype Ab (Open square, n = 7), MR1 (Open circle, n = 8), CTLA4-Ig (Open diamond, n = 7), or MR1 plus CTLA4-Ig (Open triangle, n = 8). As controls, freshly isolated islets from MRL.MpJ.Fas-/- donor mice were also transplanted to NOD mice that were treated with isotype Ab (Filled square, n = 7), MR1 (Filled circle, n = 6), or MR1 plus CTLA4-Ig (Filled triangle, n = 7). Graft survival was observed (**A**). H&E staining of kidneys harboring IPCC allografts derived from a control (**B**) or MR1+CTLA4-Ig treated recipient (**C**) was performed either at the time of graft rejection or 100 days after IPCC transplantation and survival.

To promote long-term IPCC allograft survival in diabetic NOD mice, both alloimmunity and autoimmunity must be suppressed. We determined whether T cell costimulatory blockade prevents IPCC allograft rejection in NOD recipients. IPCCs or islets derived from MRL.MpJ.Fas-/- mice (H-2^k^) were transplanted to diabetic NOD mice that were then treated with MR1 and/or CTLA4-Ig. As shown in Figure 8, a treatment with either MR1 or CTLA4-Ig significantly prolonged IPCC allograft survival (MST = 46 vs. 16 or 31 vs. 16 days, both P<0.05) although neither MR1 nor CTLA4-Ig alone induced long-term IPCC allograft survival. Importantly, combined treatments with both MR1 and CTLA4-Ig induced long-term IPCC allograft survival in six of eight NOD recipients (75%). In contrast, the combined treatments failed to prevent rejection of islet allografts in most NOD recipients (MST = 56 days, Figure 8). Moreover, blocking CD40 with MR1 was more effective in suppression of IPCC than islet allograft rejection in NOD mice (MST = 46 vs. 32 days, P<0.05). There was no cellular infiltration in IPCC grafts from MR1+CTLA4-Ig treated recipient versus massive infiltration in control grafts (Fig. 8B & 8C). These results indicate that blocking both CD40 and CD28 costimulatory pathways is required to abrogate alloimmunity and autoimmunity in NOD mice and that it is easier to suppress the rejection of ASC-derived IPCCs than islet allografts in diabetic NOD recipients.

## Discussion

Recently, several elegant studies have shown that IPCCs are generated in vitro from both human and murine ASCs [14], [15], [16], [17], suggesting that ASC-derived IPCCs may circumvent worldwide shortage of donor islets and eventually provide a cure for human type 1 diabetes. In this study, we found that ASC-derived IPCCs have the intrinsic survival disadvantage over freshly isolated islets in syngeneic recipients. Nevertheless, IPCC allografts exhibited certain immune privilege and enjoyed long-term survival in diabetic NOD mice in the presence of CD28/CD154 and Fas blockade. Therefore, long-term survival of ASC-derived IPCCs in syngeneic recipients requires only blocking Fas and TNF death pathways whereas blocking both death pathways and CD28/CD154 costimulation is needed for long-term IPCC allograft survival in diabetic NOD mice. Thus, our studies suggest that ASC-derived IPCCs may provide an effective cell replacement therapy in human type 1 diabetes.

Using IPCCs that differentiated in vitro from murine ASCs based on a recent protocol [16], we first compared their survival to that of freshly-isolated intact islets in syngeneic recipients. Surprisingly, we found that overwhelming majority of IPCC grafts did not survive for long-term in syngeneic WT or Rag1-/- mice whereas nearly all of syngeneic islet grafts survived for long-term (>100 days). Our findings suggest that freshly isolated islets have a survival advantage over in vitro-generated IPCCs upon transplantation. Therefore, IPCC syngrafts die out in vivo even in the absence of alloimmune responses, although exact mechanisms responsible for their shortened survival in syngeneic hosts remain to be defined. Our results are consistent with a previous study showing that IPCC grafts, derived from murine embryonic stem cells, have survived for only a average of two to three weeks in syngeneic Rag-/- hosts [7], implying that IPCC grafts generated from stem cells are incapable of surviving for long-term even in the absence of alloimmune-based rejection. However, our findings differ from a recent study showing that human ASC-derived IPCC grafts have survived beyond 60 days in 50% of xenogeneic recipient mice without immunosuppression [17]. It is unclear how IPCC xenografts survived for such a long time without immunosuppression.

We then investigated how to prevent IPCC graft dysfunction in the absence of alloimmunity or any immune-based rejection, which is an important step prior to in-depth IPCC allotransplantation. Since Fas or TNF signaling mediates apoptosis in most cell types, including β cells [21], [22], [23], [24], we asked whether blocking their signaling pathways would enhance IPCC graft survival or restore their function in syngeneic recipient mice. Indeed, blocking either Fas or TNF signaling significantly prolonged IPCC graft survival and inhibited IPCC cell apoptosis in syngeneic recipients while blocking both pathways induced long-term IPCC syngraft survival in overwhelming majority, if not all, of recipient mice. Our findings suggest that IPCC graft dysfunction due to passive cell death, which is mediated by Fas and TNF signal pathways but independent of alloimmune-based rejection, must also be overcome upon IPCC allotransplantation.

We next examined IPCC allograft survival in alloimmune-competent WT recipient mice. We found that IPCC allografts derived from both Fas-replete and Fas-deficient ASCs were acutely rejected in allogeneic WT recipient mice, suggesting that ASC-derived IPCCs are highly immunogenic and can evoke acute allograft rejection. Blocking CD40-CD154 costimulation induced Fas-deficient IPCC allograft survival for long-term in 63% of recipient mice. Given that the same percentage of Fas-deficient IPCC grafts survived in syngeneic recipients without CD154 costimulatory blockade, we conclude that blocking CD40-CD154 costimulation is sufficient to suppress IPCC allograft rejection mediated by alloimmune responses. Furthermore, simultaneously blocking CD154, Fas and TNF-receptor signaling induced long-term IPCC allograft survival in overwhelming majority, if not all, of recipient mice. Taken together, both death signaling of β cells and alloreactivity of recipients need to be suppressed in order to achieve long-term IPCC allograft survival.

In this study, we determined whether costimulatory blockade promotes long-term IPCC allograft survival in NOD mice that are closely relevant to human type 1 diabetes. Costimulatory blockade has been shown to suppress alloimmune responses and induce long-term allograft survival [25], [26], [27]. Previous studies have also shown that blocking both CD28 and CD154 costimulatory pathways prevents autoimmune diabetes induced by transfer of transgenic T cells in NOD.SCID mice [28] but fails to induce long-term islet allograft survival in NOD recipients [29], suggesting that CD28/CD154 blockade is sufficient to suppress autoimmunity but insufficient to simultaneously suppress both alloimmunity and autoimmunity in NOD recipients. In contrast, another study has shown that ICOS/CD154 blockade is capable of inducing islet allograft tolerance and preventing autoimmune diabetes in NOD mice [30]. Here we found that CD28/CD154 blockade induces long-term Fas-/- IPCC, but not Fas-/- islet, allograft survival in diabetic NOD recipients. We utilized Fas-deficient IPCCs because Fas-replete IPCC grafts died out over time even in syngeneic recipients due to Fas-mediated cell death. Our data have further confirmed that CD28/CD154 blockade does not induce long-term islet allograft survival in diabetic NOD recipients. On the other hand, we have demonstrated for the first time that blocking CD28 and CD154 costimulatory signaling is capable of inducing long-term survival of ASC-derived IPCC allografts in diabetic NOD mice, although a recent study has shown that blocking CD28/CD154/LFA-1 enhances engraftment of allogeneic ESC-derived endothelial cells in non-diabetic environment [31].

It is unclear whether CD154 costimulatory blockade can be translated to the clinic in the future, although it prolongs IPCC allograft survival. Clinical trials using anti-CD154 Ab were halted due to its thromboembolic side effects [32], [33]. The progress in its clinical application, hence, has been hampered. Recently, Abs against its counter receptor, CD40, have been sought as an alternative to blocking CD40/CD154 costimulatory pathway. These promising Abs have been shown to potently suppress allograft rejection in both mice and non-human primates [34], [35]. However, it remains to be defined whether they will also cause the same side effects in the future clinical trials.

Our finding indicates that it is easier to suppress the rejection of ASC-derived IPCC allografts than freshly isolated islet allografts in NOD recipient mice once passive cell death is blocked. This is consistent with previous studies showing that ESC-derived IPCCs or tissues exhibit a certain degree of immune privilege [6], [7], [36]. Therefore, ASC-derived IPCCs enjoy at least two advantages over freshly isolated islets: relatively immune privilege and unlimited sources while displaying an intrinsic survival disadvantage that is unrelated to alloimmune-based rejection. A recent study has demonstrated that transplantation of IPCCs derived from human umbilical cord mesenchymal stem cells alleviates hyperglycemia in diabetic NOD mice without any immunosuppressive treatment [37], although it was not observed how long IPCC xenografts survived and how their rejection could be suppressed. Taken together, previous and our current studies indicate that clinical application of ASC-derived IPCCs for treating type 1 diabetes is no longer elusive.

## Materials and Methods

### Ethics Statement

All animal experiments were approved by the Animal Care and Use Committee of the University of Texas Health Science Center (animal approval ID: 463B), Tyler, TX.

### Mice

Wild-type BALB/c (H-2^d^) and C57BL/6 (H-2^b^) mice were purchased from National Cancer Institute (NIH, Bethesda, MD, USA). B6.Rag1-/-, NOD, B6.MRL.Fas-/- (H-2^b^) and MRL/MpJ.Fas-/- (H-2^k^) were purchased from the Jackson Laboratory. All mice were aged 6–8 weeks when experiments were initiated. They were housed in a specific pathogen-free environment.

### Adipose-derived stem cell isolation and culture

Adipose tissues from epididymal fat and subcutaneous fat of anterior abdominal wall of donor mice were minced and digested with 0.1% type II collagenase (Invitrogen) in phosphate-buffered saline (PBS) for 30 minutes at 37°C with gentle stirring. Cell suspensions were cultured in Dulbecco's modified Eagle's medium (DMEM)/Ham's F-12 with 10% FBS and 100 U/ml penicillin, 0.1 mg/ml streptomycin (Invitrogen) at 5% CO2, 37°C. Cells were passaged under maintenance culture conditions. All experiments were performed using ASCs at 4–6 passages.

### In vitro differentiation of ASCs into IPCCs

ASCs at p4-p6 were suspended in medium M (DMEM/F12 at 1∶1) with 17.5 mM glucose, 1% BSA and 5 mg/l insulin-transferrin-selenium, plated on ultralow attachment tissue culture plates (Fisher Scientific) at 1×10^6^ cells per well, and then cultured in medium MA (M with 4 nM activin A, R&D System) for two days, medium MB (M with 0.3 mM taurine) for two days, and medium MC (M with 100 nM glucagon-like peptide-1, 1 mM nicotinamide and 1× nonessential amino acids) for five days as described previously [16]. All chemicals and supplements were purchased from Sigma Aldrich unless otherwise indicated.

### Flow cytometric analysis for phenotyping

Immunophenotyping of ASCs were performed using fluorescent-conjugated antibodies against mouse antigens MHC I (H-2K^b^), MHC II (I-A^b^), CD44, CD45, CD29, CD80, CD90, CD105 and Sca-1 (Biolegend). ASCs at the fourth passage were released by 0.25% trypsin-EDTA. A total of 1×10^6^ cells were incubated with fluorescent-conjugated Abs for 30 min at room temperature. Cells finally were analyzed using FACSCalibur.

### RT-PCR detection of pancreatic gene expression by IPCCs

Total RNA was isolated from undifferentiated ASCs, IPCCs and islets using TRIzol extraction method (TRIzol Reagent, Invitrogen). The extracted RNA was subject to cDNA synthesis using the Superscript III First-Strand Synthesis system for RT-PCR (Invitrogen). The PCR cycling conditions varied depending on different pancreatic genes and primers, which were listed in the supporting information (Table S1).

### Transplantation of islets or IPCCs

∼400 donor islets or 800 IPCCs that differentiated from ASCs were transplanted under the kidney capsule of recipient mice as described in our previous studies [38], [39]. Previous studies and our preliminary data have determined that at least 800–900 IPCCs are required to reverse hyperglycemia. Recipient mice were rendered diabetic by a single injection of streptozotocin (Sigma) (180 mg/kg for WT mice and 200 mg/kg for Rag-/- recipients) 8–10 days before transplantation. Primary graft function was defined as blood glucose under 200 mg/dl for 48 hours after transplantation. Graft rejection was defined as a rise in blood glucose to >300 mg/dl for three consecutive days after primary function. Rejection was also confirmed by histology showing cellular infiltration. In some recipients that survived for long-term, the kidney harboring IPCC grafts was removed to confirm that normalizing hyperglycemia in diabetic mice was caused by IPCC transplantation.

### Treatments of recipient mice with Abs

Recipient mice were treated with MR1 (anti-CD40L), CTLA4-Ig, or anti-TNFα Ab (Bio-Express Inc, West Lebanon, NH) at 0.25 mg on days 0, 2, 4, and 6 while anti-FasL blocking Ab (BD Biosciences) was administered at 0.1 mg on days 0, 2, 4, and 6 after transplantation.

### Histology and immunofluorescence staining

Kidney samples containing IPCC grafts from transplanted mice were harvested, fixed in 10% formalin, embedded in paraffin, sectioned with a microtome at 4 µm thickness, stained with haematoxylin/eosin (H&E), and observed for cell infiltration under the light microscope. Moreover, both IPCCs and kidneys harboring IPCC grafts were fixed, embedded and sectioned as described above. Sections were used for immunofluorescence staining of murine insulin and c-peptide. Briefly, slides were deparaffinized in xylene, rehydrated in descending grades of alcohol, immersed in citrate buffer in a microwave oven with heating for 20 min at 95°C, and treated with 0.3% hydrogen peroxide in methanol for 10 min. Sections were then placed in diluted (10%) normal serum for 20 min, covered with primary antibody (Cell Signaling) overnight at 4°C and incubated with 1∶500-diluted Alexa Fluor 488- or 568-conjugated secondary Ab solution (Invitrogen) at room temperature for 1 hour. They were finally visualized under a fluorescence microscope.

### Isolation of tissue-infiltrating cells

Tissue-infiltrating cells were isolated as described in our previous publications [40], [41]. Briefly, the kidneys harboring islet grafts were perfused *in situ* with heparinized 0.9% saline. They were then minced and digested at 37°C for 30 min in 20 ml RPMI-1640 medium containing 5% FCS and 350 u/ml collagenase (Sigma, St. Louis, MO). To clear the debris, cell suspensions were rapidly passed down 70 µm cell strainer, then mixed with Percoll solution (Sigma) to a concentration of 30%, and centrifuged at 2000 rpm for 15 minutes at room temperature. The pellet was re-suspended and stained before analysis.

### Analysis of T cell proliferation in vivo by 5-Bromo-2′-Deoxyuridine (BrdU) labeling

Recipient mice were pulsed i.p. with 0.8 mg of BrdU (Sigma) six days after transplantation. 24 hours later, renal graft-infiltrating cells were isolated and first stained with anti-CD3-PE Ab. Cells were then fixed in 70% ethanol followed by 1% paraformaldehyde and incubated with 50 Units/ml of DNase I (Sigma). Cells were finally stained with anti-BrdU-FITC (BD Biosciences) and analyzed by a FACSCalibur as described previously [40], [41].

### Analysis of insulin-producing cell apoptosis in vivo by a TUNEL method

IPCCs or islets were retrieved and dissociated with 0.25% trypsin-EDTA (Sigma-Aldrich) at 37°C for 10 minutes. To detect cell apoptosis, cells were fixed in 2% paraformaldehyde, permeabilized with 0.1% Triton X-100 solution, and labeled with fluorescein-tagged deoxyuridine triphosphate (dUTP) by the terminal deoxynucleotidyl transferase-mediated dUTP nick-end labeling (TUNEL) method according to the manufacture's instructions (Roche Applied Science, Mannheim, Germany) [40], [42].

### Statistical Analysis

Data are presented as mean ± SEM and represent an average of at least three independent experiments. Comparisons of the mean among different groups were done using the Student t test and ANOVA. The analysis of graft survival data was performed using the Kaplan-Meier method (log-rank test). A value of P<0.05 was considered statistically significant.

## Supporting Information

Figure S1 **Insulin release by IPCCs and islets in vitro.** Day-10 IPCCs or freshly isolated islets were cultured in DMEM-LG (low glucose) containing 0.5% BSA for 10 hours, washed and then stimulated or incubated in DMEM-HG (high glucose) media at 37°C for two hours. Insulin released into the media was measured using ELISA kit (Mercodia, Winston Salem, NC) according to the manufacturer's instructions. Undifferentiated ASCs were also utilized as a control. Both IPCCs and islets produced significant amount of insulin in high-glucose condition in vitro while undifferentiated ASCs did not. One of three separate experiments is shown.
(PDF)

Table S1 **Primers for RT-PCR.** The primer sequences, including the sense and anti-sense, for RT-PCR for pancreatic gene expression are listed.
(PDF)
