# Supplementary figures and images for: Promoting Long-Term Survival of Insulin-Producing Cell Grafts That Differentiate from Adipose Tissue-Derived Stem Cells to Cure Type 1 Diabetes
Source: PLoS One. 2011 Dec 28;6(12):e29706. doi: 10.1371/journal.pone.0029706 (PMC3247284; doi:10.1371/journal.pone.0029706)

**Figure S1. Insulin release by IPCCs and islets in vitro**

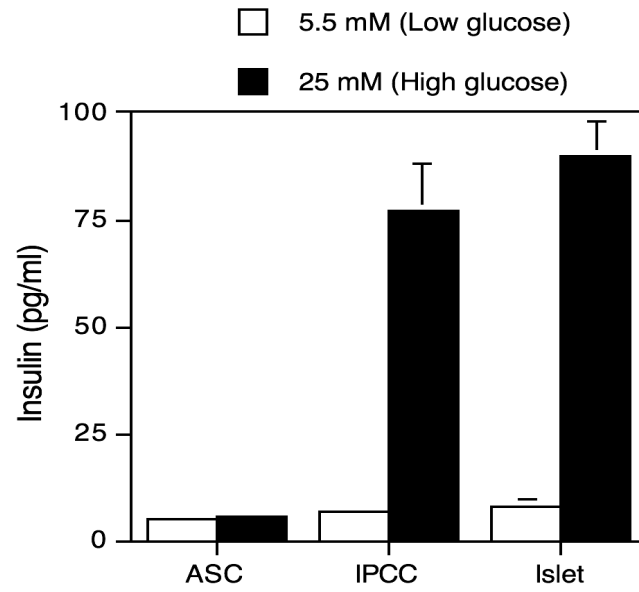

Supplement: Figure S1 — Insulin release by IPCCs and islets in vitro. Day-10 IPCCs or freshly isolated islets were cultured in DMEM-LG (low glucose) containing 0.5% BSA for 10 hours, washed and then stimulated or incubated in DMEM-HG (high glucose) media at 37°C for two hours. Insulin released into the media was measured using ELISA kit (Mercodia, Winston Salem, NC) according to the manufacturer's instructions. Undifferentiated ASCs were also utilized as a control. Both IPCCs and islets produced significant amount of insulin in high-glucose condition in vitro while undifferentiated ASCs did not. One of three separate experiments is shown. (PDF) [file pone.0029706.s001.pdf]
