# Supplementary material for: Promoting Long-Term Survival of Insulin-Producing Cell Grafts That Differentiate from Adipose Tissue-Derived Stem Cells to Cure Type 1 Diabetes
Source: PLoS One. 2011 Dec 28;6(12):e29706. doi: 10.1371/journal.pone.0029706 (PMC3247284; doi:10.1371/journal.pone.0029706)
Supplement: Table S1 — Primers for RT-PCR. The primer sequences, including the sense and anti-sense, for RT-PCR for pancreatic gene expression are listed. (PDF) [file pone.0029706.s002.pdf]

Table S1: Primers for RT-PCR

No.	Name	Sequence 5'--3'	Product size
1	PDX-1	ACTTAACCTAGGCGTCGCACAAGA	135bp
		GGCATCAGAAGCAGCCTCAAAGTT	
2	GCK	ACAGAGCCAGGATGGAGG	127bp
		TTCAGGCCACGGTCCA	
3	ISL1	ACAAGCGGTGCAAGGACAAGAAAC	154bp
		ACTGGGTTAGCCTGTAAACCACCA	
4	PAX4	TGTTACAAGACCAGACCACCAGCA	190bp
		TGCATGCTTCACACTGGTACTCCT	
5	Glut2	TGTCATCGCCCTCTGCTTC	161bp
		TCTTCCGGAATTCTGCAGC	
6	NeuroD1	ACCTTGCTACTCCAAGACCCAGAA	139bp
		TTTGCAGAGCGTCTGTACGAAGGA	
7	SST	ATGCTGTCCTGCCGTCTC	127bp
		TGGCAGCCGCCAGAG	
8	Insulin	TGGCTTCTTCTACACACCCAAG	131bp
		ACAATGCCACGCTTCTGCC	
9	β-actin	AGGTCATCACTATTGGCAACGA	118bp
		CACTTCATGATGGAATTGAATGTAGTT	
